# Supplementary material for: OpenMM 7: Rapid development of high performance algorithms for molecular dynamics
Source: PLoS Comput Biol. 2017 Jul 26;13(7):e1005659. doi: 10.1371/journal.pcbi.1005659 (PMC5549999; doi:10.1371/journal.pcbi.1005659)
Supplement: S1 Source Code — (ZIP) [file pcbi.1005659.s002.zip › OpenMM-7.0.1-Source/docs/api-c++/generated/OpenMM.AmoebaGeneralizedKirkwoodForce.html]

AmoebaGeneralizedKirkwoodForce — OpenMM @OPENMM\_MAJOR\_VERSION@.@OPENMM\_MINOR\_VERSION@ documentation


# AmoebaGeneralizedKirkwoodForce¶

*class* `OpenMM::``AmoebaGeneralizedKirkwoodForce`¶
:   This class implements an implicit solvation force using the generalized Kirkwood/Grycuk model.

    To use this class, create an `AmoebaGeneralizedKirkwoodForce` object, then call `addParticle()` once for each particle in the `System` to define its parameters. The number of particles for which you define parameters must be equal to the number of particles in the `System`, or else an exception will be thrown when you try to create a `Context`. After a particle has been added, you can modify its force field parameters by calling `setParticleParameters()`. This will have no effect on Contexts that already exist unless you call `updateParametersInContext()`.

    Methods

    |  |  |
    | --- | --- |
    | `AmoebaGeneralizedKirkwoodForce` |  |
    | `getNumParticles` | Get the number of particles in the system. |
    | `addParticle` | Add the parameters for a particle. |
    | `getParticleParameters` | Get the force field parameters for a particle. |
    | `setParticleParameters` | Set the force field parameters for a particle. |
    | `getSolventDielectric` | Get the dielectric constant for the solvent. |
    | `setSolventDielectric` | Set the dielectric constant for the solvent. |
    | `getSoluteDielectric` | Get the dielectric constant for the solute. |
    | `setSoluteDielectric` | Set the dielectric constant for the solute. |
    | `getIncludeCavityTerm` | Get the flag signaling whether the cavity term should be included |
    | `setIncludeCavityTerm` | Set the flag signaling whether the cavity term should be included |
    | `getProbeRadius` | Get the probe radius (nm) used in SASA contribution |
    | `setProbeRadius` | Set the probe radius (nm) used in SASA contribution |
    | `getSurfaceAreaFactor` | Get the surface area factor kJ/(nm\*nm) used in SASA contribution |
    | `setSurfaceAreaFactor` | Set the surface area factor kJ/(nm\*nm) used in SASA contribution |
    | `updateParametersInContext` | Update the per-particle parameters in a `Context` to match those stored in this `Force` object. |
    | `usesPeriodicBoundaryConditions` | Returns whether or not this force makes use of periodic boundary conditions. |

    `AmoebaGeneralizedKirkwoodForce`()¶

    int `getNumParticles`() *const*¶
    :   Get the number of particles in the system.

    int `addParticle`(double *charge*, double *radius*, double *scalingFactor*)¶
    :   Add the parameters for a particle. This should be called once for each particle in the `System`. When it is called for the i’th time, it specifies the parameters for the i’th particle.

        |  |  |
        | --- | --- |
        | Parameters: |  |

        - **charge** – the charge of the particle, measured in units of the proton charge
        - **radius** – the atomic radius of the particle, measured in nm
        - **scalingFactor** – the scaling factor for the particle

        |  |  |
        | --- | --- |
        | Returns: | the index of the particle that was added |

    void `getParticleParameters`(int *index*, double &*charge*, double &*radius*, double &*scalingFactor*) *const*¶
    :   Get the force field parameters for a particle.

        |  |  |
        | --- | --- |
        | Parameters: |  |

        - **index** – the index of the particle for which to get parameters
        - **charge** – [out] the charge of the particle, measured in units of the proton charge
        - **radius** – [out] the atomic radius of the particle, measured in nm
        - **scalingFactor** – [out] the scaling factor for the particle

    void `setParticleParameters`(int *index*, double *charge*, double *radius*, double *scalingFactor*)¶
    :   Set the force field parameters for a particle.

        |  |  |
        | --- | --- |
        | Parameters: |  |

        - **index** – the index of the particle for which to set parameters
        - **charge** – the charge of the particle, measured in units of the proton charge
        - **radius** – the atomic radius of the particle, measured in nm
        - **scalingFactor** – the scaling factor for the particle

    double `getSolventDielectric`() *const*¶
    :   Get the dielectric constant for the solvent.

    void `setSolventDielectric`(double *dielectric*)¶
    :   Set the dielectric constant for the solvent.

    double `getSoluteDielectric`() *const*¶
    :   Get the dielectric constant for the solute.

    void `setSoluteDielectric`(double *dielectric*)¶
    :   Set the dielectric constant for the solute.

    int `getIncludeCavityTerm`() *const*¶
    :   Get the flag signaling whether the cavity term should be included

    void `setIncludeCavityTerm`(int *includeCavityTerm*)¶
    :   Set the flag signaling whether the cavity term should be included

    double `getProbeRadius`() *const*¶
    :   Get the probe radius (nm) used in SASA contribution

    void `setProbeRadius`(double *probeRadius*)¶
    :   Set the probe radius (nm) used in SASA contribution

    double `getSurfaceAreaFactor`() *const*¶
    :   Get the surface area factor kJ/(nm\*nm) used in SASA contribution

    void `setSurfaceAreaFactor`(double *surfaceAreaFactor*)¶
    :   Set the surface area factor kJ/(nm\*nm) used in SASA contribution

    void `updateParametersInContext`(Context &*context*)¶
    :   Update the per-particle parameters in a `Context` to match those stored in this `Force` object. This method provides an efficient method to update certain parameters in an existing `Context` without needing to reinitialize it. Simply call `setParticleParameters()` to modify this object’s parameters, then call `updateParametersInContext()` to copy them over to the `Context`.

        The only information this method updates is the values of per-particle parameters. All other aspects of the `Force` (the probe radius, the surface area factor, etc.) are unaffected and can only be changed by reinitializing the `Context`.

    bool `usesPeriodicBoundaryConditions`() *const*¶
    :   Returns whether or not this force makes use of periodic boundary conditions.

        |  |  |
        | --- | --- |
        | Returns: | true if nonbondedMethod uses PBC and false otherwise |

High performance molecular simulation on GPUs


### Navigation

- Core Objects
- Forces
- Integrators
- Extras

©2015, Stanford University and the Authors.
|
Powered by Sphinx 1.3.1
& Alabaster 0.7.7
|
Page source
